# Client-specific outcome measure for chronic osteoarthritis pain assessment in horses

**DOI:** 10.3389/fvets.2026.1771745

**Published:** 2026-03-23

**Authors:** Eleonora Benetti, Adolfo Maria Tambella, Sabrina Nathalie Andreis, Stefan Witte, Caterina Di Bella, Claudia Spadavecchia

**Affiliations:** 1 Vetsuisse Faculty, Department of Clinical Veterinary Medicine, Section of Anesthesiology and Pain Therapy, University of Bern, Bern, Switzerland; 2 School of Biosciences and Veterinary Medicine, University of Camerino, Camerino, Matelica, Italy; 3 Amt für Veterinärwesen, Bern, Switzerland; 4 Tierklinik Schönbühl AG, Schönbühl, Switzerland

**Keywords:** chronic pain, client specific outcome measures, CSOM, equine, osteoarthritis, subjective assessment

## Abstract

**Introduction:**

Osteoarthritis (OA) is a prevalent cause of chronic pain and lameness in horses. Whereas lameness can be quantified using objective measures, the assessment of OA-associated pain remains challenging. This study aimed to evaluate the applicability of the Client-Specific Outcome Measure (CSOM), a tool widely used in small animals, for the assessment of chronic OA pain in horses through caretaker evaluation.

**Methods:**

Seventeen privately owned horses with confirmed OA were enrolled in a 20-week randomized, double-blind, placebo-controlled study. For each horse, three individual pain-related indicators (CSOM items) were identified through a veterinarian–caretaker consultation. The selected items were regularly scored (0–4), and the scores were summed to obtain a total CSOM score (CSOM-sum). The CSOM-sum was compared with other pain assessment measures, including a caretaker-assigned visual analogue scale (VAS-own), a veterinarian-assigned visual analogue scale (VAS-exp), subjective lameness scores, and a gait asymmetry index.

**Results:**

The CSOM-sum showed moderate but significant correlations with all other pain and lameness measures (*rₛ* = 0.49–0.60, *p* < 0.05). In particular, CSOM-sum correlated with gait asymmetry (rₛ = 0.434, *p* < 0.0001); subgroup analysis revealed a moderate correlation in treated horses (*rₛ* = 0.4539, *p* = 0.0025) and a higher correlation in controls (*rₛ* = 0.5536, *p* = 0.0006). The VAS-own and VAS-exp scores showed good overall agreement (bias = −4.76 mm; ICC = 0.727; ICC_T = 0.625; ICC_C = 0.838), although with relatively wide limits of agreement. The internal consistency of the CSOM items was high (Cronbach’s *α* = 0.81 overall; α_T = 0.787; α_C = 0.890).

**Discussion:**

These findings indicate that, provided relevant items are carefully identified, the CSOM may represent a valuable complementary tool for assessing and monitoring pain severity in horses under field conditions.

## 1 Introduction

Osteoarthritis (OA) is a common and progressive condition that affects humans and animals. In horses, it can manifest at any age, from foals to adults, with an incidence that can reach up to 66% (1–3). OA causes reluctance to move, lameness, and chronic pain, often leading to early retirement from competition and significant economic losses for owners. Assessing pain and disability in affected horses represents a challenge (3, 4). Unlike humans, horses cannot verbally communicate their symptoms, making the development of effective and reliable pain assessment methods crucial. Veterinarians traditionally rely on physical examinations, gait analysis, and diagnostic imaging to evaluate the severity of OA and to monitor treatment responses (5). However, these methods may not fully capture the subjective experience of pain and its impact on the horse’s daily life. In recent years, research on equine chronic pain has introduced more specific assessment instruments, such as the Musculoskeletal Pain Scale (MPS), the Horse Chronic Pain Scale (HCPS) and the Equine Brief Pain Inventory (EBPI) (6–8). Based on behavioral and physiological indicators, these scales aim to provide more objective and standardized evaluation of pain severity. Despite these advances, fully capturing the individual impact of pain is still a challenge (7–9). To address this limitation, the importance of including owners and caretakers in the evaluation of a horses’ wellbeing is increasingly recognized, as has long been the case with companion animals. Caretakers’ involvement is crucial because, through daily observation, it allows the detection of subtle changes that might go unnoticed during a veterinary examination (10, 11). Consequently, there is growing interest in using Client-Specific Outcome Measures (CSOM), a concept adapted from human medicine where Patient-Reported Outcome Measures (PROMs) are used (12–14). In contrast to standardized questionnaires, CSOM are customized for each individual animal, allowing caretakers to identify and assess specific activities that may be compromised by pain or disability. These tools have proven useful in evaluating chronic pain in companion animals, highlighting improvements in mobility after pharmacological treatments or surgical interventions, and bringing significant contributions to the assessment of quality of life. The involvement of caretakers in assessing the clinical condition of horses has only recently received greater attention, particularly regarding lameness evaluation through standardized questionnaires (11). Despite some limitations, previous studies have shown that caretakers’ assessments, while sometimes underestimating or struggling to detect signs of pain, are often consistent with expert evaluations (10).

The aim of the present study was to evaluate the usefulness of CSOM as an additional tool for assessing and monitoring chronic pain in horses affected by osteoarthritis (OA). Specifically, the study investigated the characteristics and categories of CSOM and the correlations between CSOM scores and other pain assessment measures, including visual analogue scale (VAS) scores assigned by both caretakers (VAS-own) and veterinarians (VAS-exp), subjective lameness scores, and the gait asymmetry index. While VAS scores provide a subjective assessment of pain severity, lameness scores and the gait asymmetry index primarily evaluate lameness, which is used as an indirect indicator of pain.

## 2 Materials and methods

### 2.1 Study cohort

Seventeen private-owned adult horses (9 mares and 8 geldings) affected by spontaneous osteoarthritis and showing signs of chronic musculoskeletal pain were recruited within the context of a longitudinal clinical trial investigating a novel analgesic therapy (not published yet). Horses had a mean age of 17.2 years (range 8–29 years) and body weight of 575.9 kg (range 430–670 kg) and remained under owner’s care in their usual environment for the duration of the study. Owners signed an informed written consent and a permission to perform the study was obtained from the Cantonal Committee for Animal Experiments of the Canton of Bern, Switzerland (Permission number: 32026).

### 2.2 Inclusion and exclusion criteria

An active recruitment campaign to find horses with a pre-existing diagnosis of appendicular osteoarthritis was conducted between March and June 2020. The inclusion criteria required horses to be over 3 years of age and to have a history of single-limb lameness lasting at least 3 months, along with clinical signs consistent with osteoarthritis—such as an abnormal or stiff gait, difficulty lying down or rising, reduced overall performance, or intolerance to being ridden. Radiographic or MRI evidence was required to confirm osteoarthritis in at least one appendicular joint. Exclusion criteria included recent therapy with NSAIDs (within the last 2 weeks), corticosteroids (within the last 4 weeks), or the presence of systemic diseases and/or neurological disorders.

To ensure the absence of other clinical abnormalities, a thorough clinical and neurological examination was performed, and blood samples were collected for hematology and biochemistry profiles. Seventy-two horse owners initially responded to the recruitment campaign, and 20 horses were considered potentially eligible. After a complete clinical examination, 17 horses fully met the inclusion criteria and were recruited for the study over a period of 20 weeks. Fifteen horses completed the study. Two horses were euthanized due to intractable lameness at 2 months (H6) and 4 months (H2) after enrollment, respectively. Both horses belonged to the control group (C).

### 2.3 Study design

The present investigation focused on data collected within the context of a larger prospective, double-blind, placebo-controlled clinical field trial. While the primary study was designed to evaluate the efficacy of an analgesic treatment compared with placebo (results not published yet), the aim of the present analysis was to evaluate the applicability and performance of the CSOM as an outcome tool. Within the framework of the primary study, horses were randomly assigned to either group T (treatment; *n* = 8) or group C (placebo; *n* = 9), according to a computer-generated randomization sequence.^1^ Treatment or placebo (1 mL) was administered subcutaneously three times at 4-week intervals over a total study period of 20 weeks. Both the caretakers and the veterinarian responsible for all the clinical assessments (SA) were blinded to treatment allocation.

### 2.4 Definition of individual CSOM

**Figure 1:**
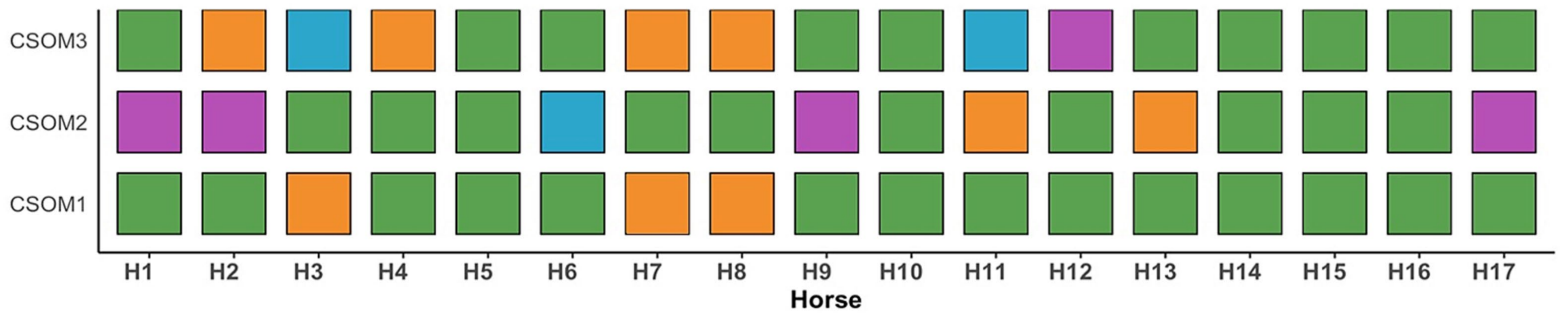
Distribution of CSOM behavioral indicators across individual horses. Each column represents one horse (H1–H17). For each horse, three equally sized squares are shown, corresponding to the three CSOM indicators recorded during the assessment (CSOM1–CSOM3). Each square represents a single observed CSOM indicator, regardless of category. Square color indicates the CSOM category: facial expression (light blue), evoked pain reaction (light purple), motivation and activity (light orange), and posture and weight-bearing (light green). Squares are displayed even when multiple indicators belong to the same category, allowing direct visual comparison of the three CSOM indicators within each horse. The figure illustrates the distribution of CSOM indicator categories per horse and does not represent frequencies or percentages.

During the initial screening visit (T0), caretakers were asked to identify three main concerns, behaviors or symptoms that they observed in their horse and that were perceived as reflecting OA severity. These could be, for example lameness at the walk, pain upon palpation of the affected joint, difficulty walking downward, or reluctance to move. Each selected item was designated as CSOM1, CSOM2, or CSOM3, respectively, as listed in Supplementary materials (Supplementary Table S1). A single experimenter (SA) was responsible for interviewing the horse caretaker, assisting with the definition of the CSOM and preparing *ad hoc* sheets for subsequent data collection. The order of the CSOM on the data collection sheet was randomized. Caretakers were instructed to consistently use the initially defined CSOM throughout the study, with no alterations permitted. They were then required to score each CSOM on a scale from 0 to 4, where 0 indicated absence of the symptom/no pain and 4 indicated severe expression of the symptom/maximal pain (Figure 1). For each horse, CSOM were assessed always by the same caretaker on a weekly basis, with each assessment referring to observations made during the preceding week. Only data collected at 4, 8, 12, 16, and 20 weeks (T1, T2, T3, T4, and T5, respectively) after the start of treatment were included in the specific analysis, as veterinary examinations and objective gait analysis were performed exclusively at these time points.

### 2.5 Visual analogue scale (VAS), lameness score, and gait asymmetry index

During the initial clinical examination and at each subsequent assessment every 4 weeks, pain was assessed using a visual analogue scale (VAS). Both the veterinarian (SA) and the horse caretaker, who were blinded to the treatment allocation, performed this assessment independently, by marking the perceived pain severity on a 100 mm horizontal line, where 0 mm represented no pain and 100 mm the worst possible pain. Based on previous studies, reference values for pain classification were adopted, with 0–4 mm corresponding to no pain, 5–44 mm to mild pain, 45–74 mm to moderate pain and 75–100 mm to severe pain (15). Lameness severity was assessed applying both subjective and objective methods. An experienced equine veterinarian (SA) assessed lameness severity on the 0–10 UK lameness scale where 0 indicates that the horse is clinically sound, and 10 corresponds to a non-weight bearing lameness (16, 17). The observation was conducted by assessing the horse at the walk and trot on a hard, straight surface, as well as on a circle at walk. Subsequently, a validated system consisting of inertial measurement units (Xsens Technologies B.V., Enschede, The Netherlands) and customized software (EquiGait Ltd., Cheshunt, Herts, UK) was used to quantify gait asymmetry. The collected data were processed as described in previous studies (18–20). Lameness was defined as MnD (difference between the vertical minima reached during left and right stance) and/or MxD (difference between the vertical maxima reached during left and right stance) greater than 6 mm for the forelimb and 3 mm for the hindlimb, based on previously established references. As a gait asymmetry index, the vector sum (VS) was calculated from poll (forelimb lameness) and pelvis (hindlimb lameness) acceleration data as previously described: VS = √((MnD × MnD) + (UpD × UpD)). UpD = difference between the vertical movement amplitudes from the minimum at mid stance to the maximum in the aerial phase between the left and right half of the stride cycle (21).

## 3 Statistical analysis

For statistical analysis, the scores attributed to each CSOM item for an individual horse at a certain time point were summed to obtain a CSOM-Sum. The relationship between CSOM-Sum, VAS-own, VAS-exp, lameness score, and gait asymmetry index was analyzed by Spearman’s correlation and quantified with the *rₛ* coefficient in a correlation matrix. The *rₛ* coefficient was considered significant if it was greater than the critical value of *rₛ* for *p* < 0.05. In addition, the relationship between the CSOM-Sum and Gait Asymmetry Index was analyzed using Spearman’s correlation (*rₛ*) with the Naïve approach, which assumes independent observations. Agreement between VAS-own and VAS-exp was also quantified by the Bland–Altman method, calculating the mean bias and standard deviation of the bias. Calculation of the 95% confidence interval (95% CI) allowed determination of the limit of inter-rater agreement (LOA). To evaluate data dispersion, Bland–Altman (mean/difference) scatter plots were generated. Inter-rater reliability between the VAS-caretaker and VAS-examiner was analyzed using the intraclass correlation coefficient (ICC) and 95% CI. In accordance with the experimental design and conceptual intent of the study, the ICC was calculated using a two-way random-effects, absolute agreement, single-rater model (ICC (2,1)), following the convention established by Shrout and Fleiss (22) and later refined by Koo and Li (23). As the values of the ICC range from 0 to 1, they were interpreted as follows: values below 0.4 indicate poor reliability, between 0.4 and 0.75 moderate reliability, between 0.75 and 0.9 good reliability, and above 0.9 excellent reliability (22, 24). In addition to the ICC, standard error of measurement (SEM), minimum detectable change at 95 percent (MDC), and percent of MDC (%MDC) were calculated as measurement errors of reliability parameters. The internal consistency of the three CSOM items was assessed by calculating the Cronbach’s alpha coefficient, which was interpreted as follows: values below 0.4 indicate low consistency, between 0.4 and 0.6 moderate consistency, between 0.6 and 0.8 acceptable/adequate consistency, between 0.8 and 0.9 good consistency, and above 0.9 verification of potential redundancy. All data were analyzed both by group and in the entire population using GraphPad Prism 9 software for macOS, version 9.3.1 (GraphPad Software Inc., San Diego, CA, USA) or Microsoft Excel for Mac (version 16.66.1, Microsoft 365, 2022) extended with Real Statistics Resource Pack software (Release 8.4).

## 4 Results

For a descriptive purpose, the CSOM items identified by the horse caretakers were categorized into four domains: Posture and weight bearing (66.7%), Motivation and activity (17.6%), Evoked pain response (9.8%), and Facial expression (5.9%). A graphical representation of the category’s distribution is presented in Figure 1.

**Figure 2:**
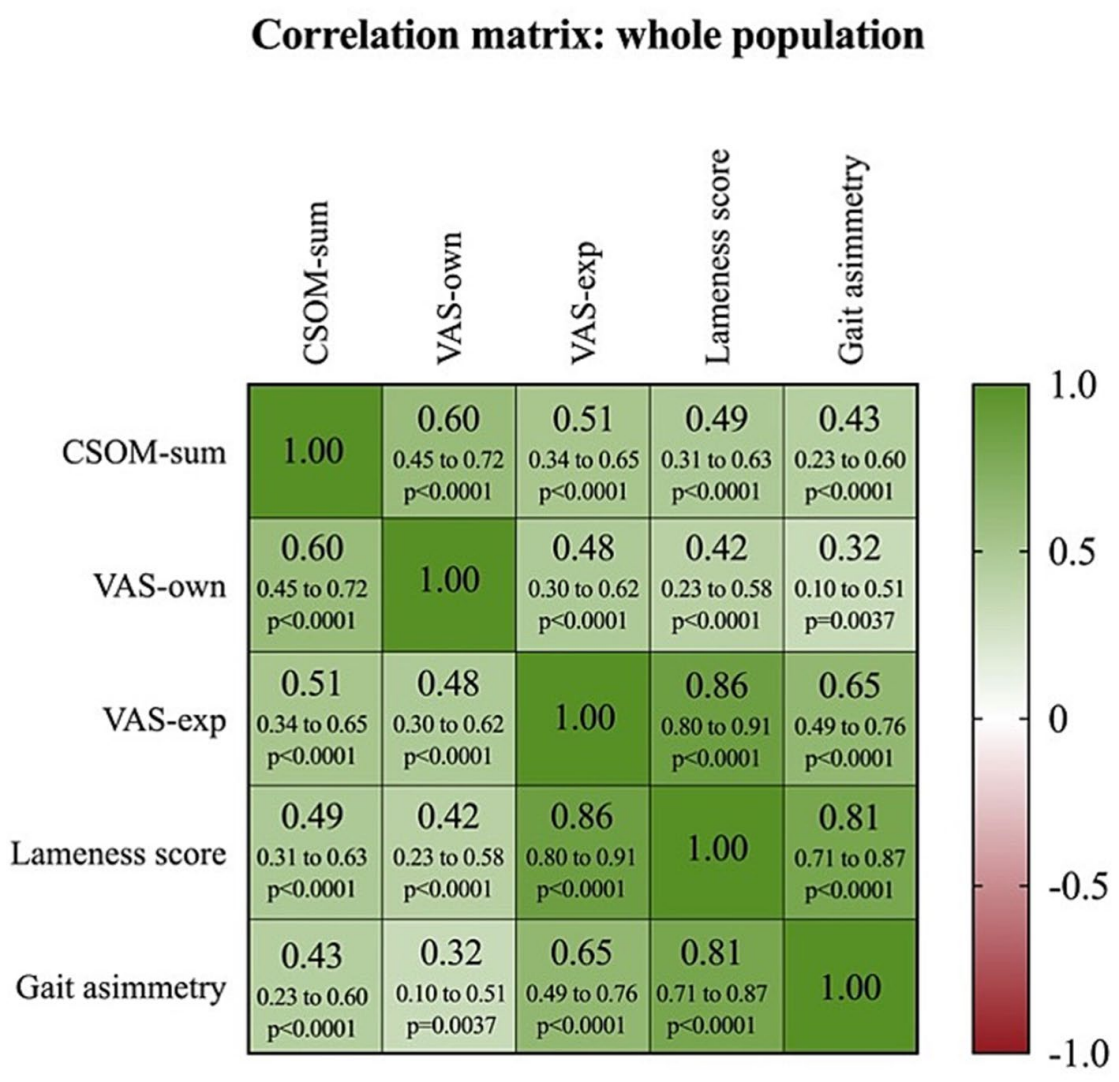
Heat map of correlation matrix showing the relationship between Client-Specific Outcomes Measure (CSOM-sum), Visual Analogue Scale by caretakers (VAS-own), Visual Analogue Scale by examiner (VAS-exp), lameness score, and gait asymmetry index, considering the whole population. Different shades of green indicate a positive correlation, while different shades of red indicate a negative correlation. In each cell, the Spearman correlation coefficient (*r_s_*), the 95% confidence intervals (95%CI) and the *p*-value (*p*) are shown on the first, second and third lines, respectively, (critical value of *r_s_* = 0.189).

**Figure 3:**
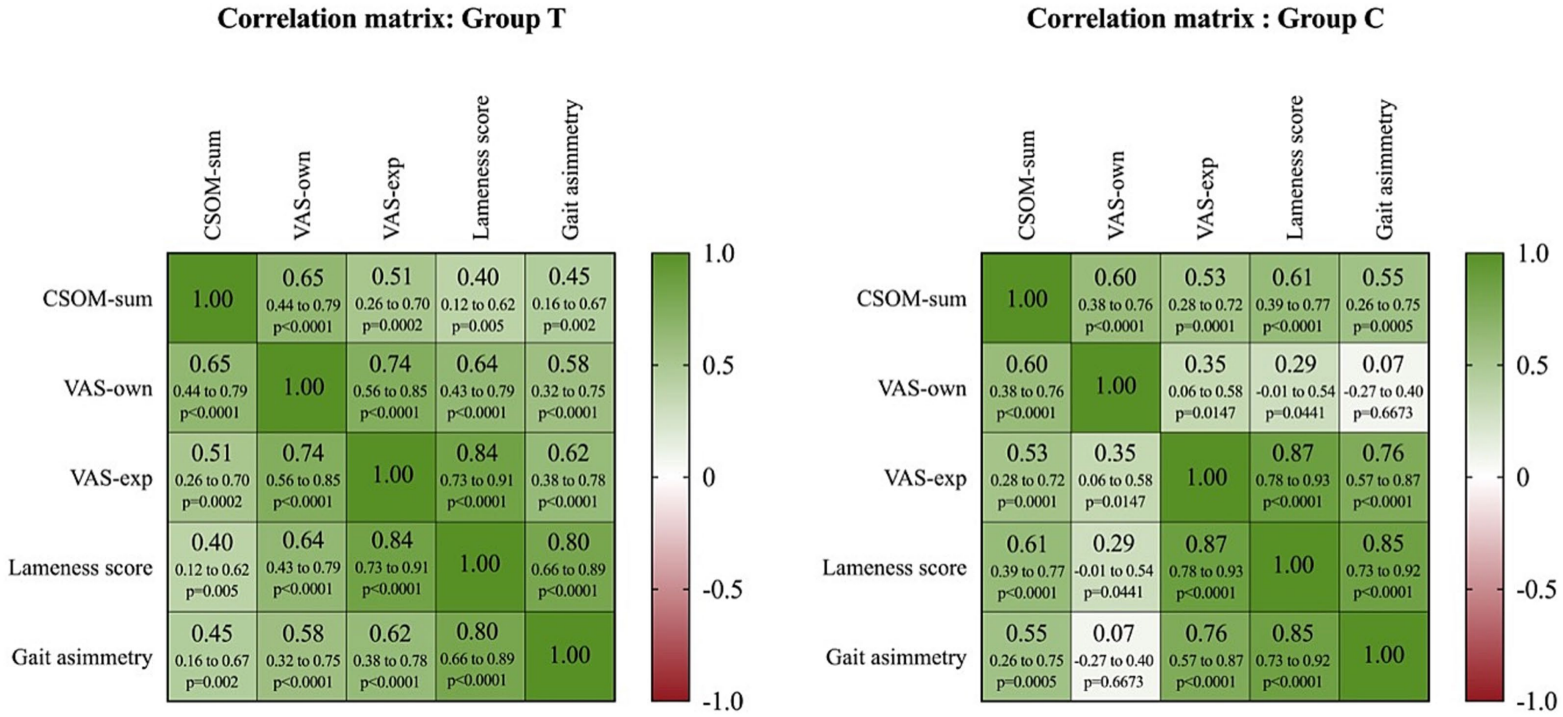
Heat map of correlation matrix showing the relationship between Client-Specific Outcomes Measure (CSOM), Visual Analogue Scale by owner (VAS-own), Visual Analogue Scale by examiner (VAS-exp), and lameness score, considering Group T and Group C. Different shades of green indicate a positive correlation, while different shades of red indicate a negative correlation. In each cell, the Spearman correlation coefficient (*rₛ*), the 95% confidence intervals (95% CI), and the *p*-value (*p*) are shown on the first, second, and third lines, respectively, (critical value of *rₛ* = 0.257 and *rₛ* = 0.283 in Group T and C respectively).

Spearman’s correlation showed a significant relationship of CSOM-sum to VAS-own, VAS-exp, lameness score and gait asymmetry index (*p* < 0.05), both in the whole sample (Figure 2) and in the two study groups (T and C, respectively) (Figure 3).

An additional analysis of the relationship between CSOM-Sum and Gait Asymmetry Index revealed a moderate positive correlation in the total population (T + C) (*rₛ* = 0.4340, 95% CI: 0.2262–0.6040, *p* < 0.0001) (Figure 2), indicating that a higher CSOM-Sum is associated with a higher degree of gait asymmetry. When analyzed separately, the correlation remained moderate in the T group (*rₛ* = 0.4539, 95% CI: 0.1650–0.6711, *p* = 0.025) and was stronger in the C group (*rₛ* = 0.5536, 95% CI: 0.2607–0.7532, *p* = 0.0006), suggesting a stronger relationship in this subgroup of horses (Table 1).

**Table 1:** Agreement evaluation for visual analogue scale (VAS).

Groups/population	VAS-exp	VAS-own	Bland–Altman (B&A) method		
	Mean ± sd	Mean ± sd	Bias	Sd of bias	LOA (95%CI)
Whole population	33.03 ± 22.44	38.19 ± 20.29	−4.760	20.98	−45.87 to 36.35
Group T	30.88 ± 22.20	36.83 ± 16.98	−5.958	15.73	−36.80 to 24.88
Group C	35.19 ± 22.70	39.51 ± 23.19	−3.563	25.28	−53.11 to 45.98

VAS-exp, Visual Analogue Scale by examiner; VAS-own, Visual Analogue Scale by owner; sd, standard deviation; LOA, limits of agreement; 95% CI, 95% confidence interval.

**Figure 4:**
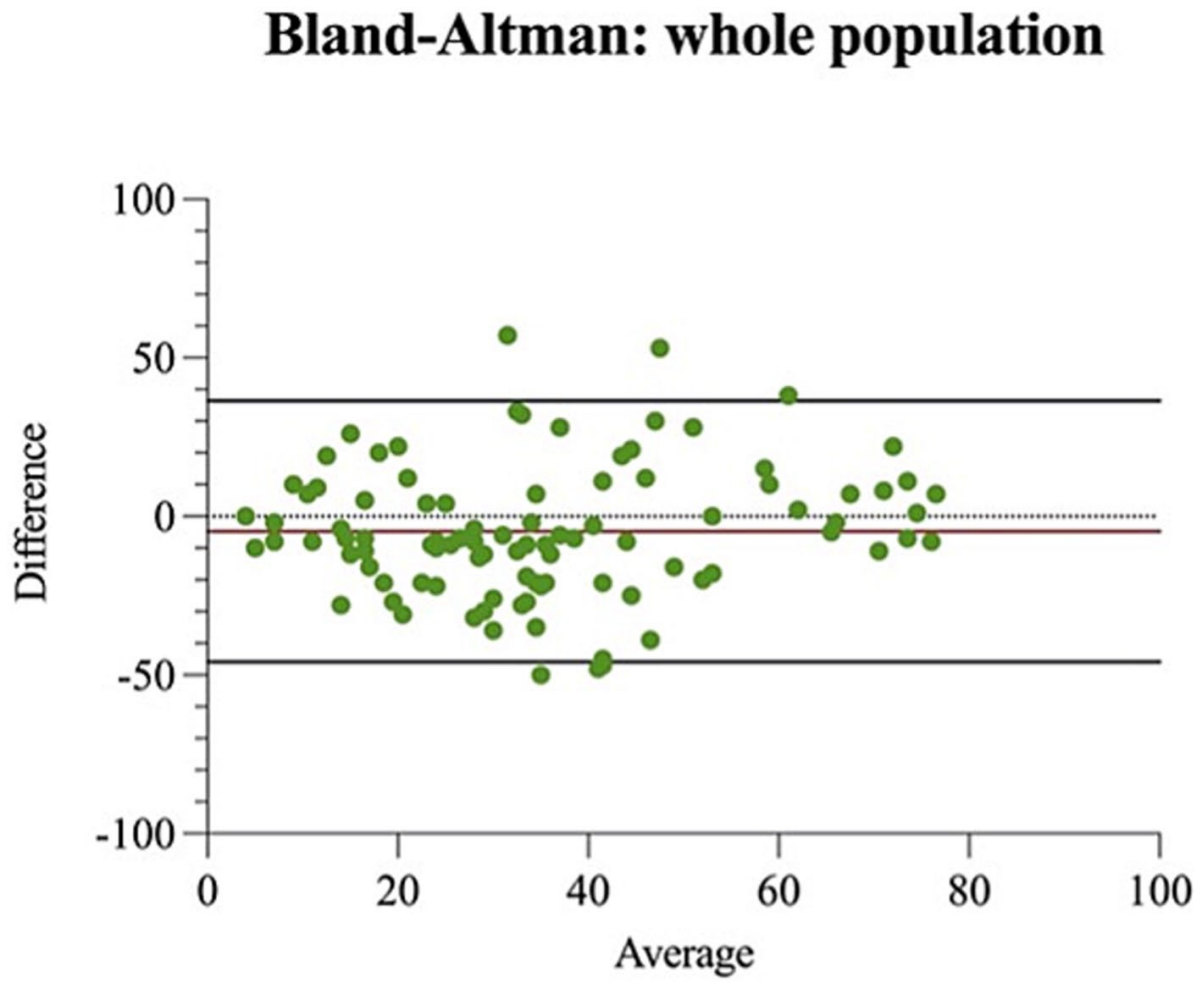
Bland–Altman scatter plot (average/difference) of visual analogue scale performed by the examiner (VAS-exp) and the owner (VAS-own) in the whole population, with bias (red line) and 95% CI limits of agreement (black lines). The dotted line at *y* = 0 indicates perfect agreement.

A good level of agreement between VAS-own and VAS-exp emerged from the Bland–Altman analysis (Figure 4).

Furthermore, the inter-rater reliability for VAS-own and VAS-exp was good on whole-sample analysis (ICC = 0.727, with a SEM of 11.73). The group-level analysis showed moderate and good reliability, with ICC values of 0.625 and 0.838, respectively (Table 2).

**Table 2:** Inter-rater reliability evaluation for visual analogue scale (VAS).

Groups/Population	Inter-rater reliability between VAS-exp and VAS-own				
	ICC	95%CI ICC	SEM	MDC	MDC%
Whole population	0.727	0.594 to 0.816	11.732	32.521	98.454
Group T	0.625	0.398 to 0.776	13.600	37.697	122.097
Group C	0.838	0.723 to 0.906	9.124	25.289	71.870

VAS-exp, Visual Analogue Scale by examiner; VAS-own, Visual Analogue Scale by owner; ICC, Intraclass Correlation Coefficent; 95%CI ICC, 95% confidence interval of ICC; SEM, standard error of measurement; MDC, minimal detectable change at 95%; %MDC, percentage of MDC.

The evaluation of the internal consistency of the three CSOM items showed good consistency (alpha = 0.809) in the whole sample and in group C (alpha = 0.890), and adequate consistency in group T (alfa = 0.787) (Table 3).

**Table 3:** Internal consistency evaluation for client-specific outcomes measure (CSOM).

Groups/Population	Cronbach’s alpha coefficient	Cronbach’s alpha coefficient with missing item		
		CSOM 1	CSOM 2	CSOM 3
Whole population	0.80950128	0.65615788	0.81089331	0.59856744
Group T	0.78684125	0.67416602	0.83557335	0.59746328
Group C	0.89035886	0.73562269	0.82464592	0.73319211

CSOM, Client-Specific Outcomes Measure; CSOM 1, 2, 3: CSOM item #1, #2, #3, respectively.

## 5 Discussion

The distribution of CSOM items revealed that most observations (66.7%) were related to posture and weight-bearing, followed by motivation and activity (17.6%), evoked pain responses (9.8%), and facial expressions (5.9%). This pattern suggests that caretakers tend to recognize postural and locomotor changes earlier and more consistently than more subtle indicators of chronic pain, such as facial tension or specific reactive behaviors. Similar findings have been described in other species, where overt motor alterations are among the first and most salient signs detected by non-professional observers (25, 26). The lower frequency of facial and evoked response items highlights the need to promote caretaker education on recognizing less apparent behavioral signs of discomfort, which may improve the sensitivity of owner-based outcome measures.

The main objective of this study was to evaluate the effectiveness of client-specific outcome measures (CSOM) in assessing chronic osteoarthritis pain in horses. The results support the hypothesis that CSOM may be a valid tool for assessing chronic pain in this species. Correlation analysis showed that the CSOM-Sum total score was moderately associated with VAS-own, VAS-exp and lameness scores, confirming that CSOM may effectively reflect a subjectively perceived component of pain in horses. In particular, the correlation between CSOM-sum and gait asymmetry index showed that higher CSOM-sum scores are associated with a higher degree of gait asymmetry. This association was significant both in the entire population (*rₛ* = 0.434, *p* < 0.0001) and in the two subpopulations analyzed separately, with a moderate correlation in group T (*rₛ* = 0.4539, *p* = 0.025) and a stronger correlation in group C (*rₛ* = 0.5536, *p* = 0.0006). This suggests that CSOM could complement biomechanical assessments to provide a more comprehensive picture of the animal’s clinical condition. A further analysis focusing on the relationship between the VAS-own and gait asymmetry revealed a moderate and significant correlation in the treated group (T) (*rₛ* = 0.58; p < 0.0001), but a low and non-significant correlation in the control group (C) (*rₛ* = 0.27; *p* = 0.0667). Since the caretakers were unaware of the treatment assignment, this difference cannot be attributed to the expectation of bias. In treated horses, improvements in locomotor function and pain reduction likely occurred in parallel, allowing caretakers to detect changes that correspond to objective measurements of asymmetry. In untreated horses, the clinical status tended to remain stable or deteriorate slowly, with fluctuations in pain and asymmetry that did not always progress together. In chronic cases, compensatory gait adaptations may reduce asymmetry without alleviating pain (or vice versa), contributing to a weaker association between subjective and objective measures. These findings highlight the need to combine subjective owner assessments with objective measures, particularly in untreated subjects, to ensure accurate clinical monitoring.

Furthermore, the good level of agreement between caretakers and examiners suggests that caretaker involvement in equine pain assessment can be a key element for long-term monitoring. These results are also consistent with previous studies in dogs and cats with osteoarthritis, in which CSOM compared with other objective measures, such as Activity Monitoring (AM) in cats and Pressure Contact Variables (PCV) or Force Plate Analysis in dogs, proved to be a reliable method for monitoring pain progression and treatment efficacy (26–28). However, it is important to clarify to be made is that while CSOMs in small animal studies were compared with validated instruments, this was not possible in horses due to the lack of standardized reference scales for chronic pain. This limits the possibility of direct comparisons and underlines the need for further studies to validate the CSOM in the equine species. However, there are notable parallels between the present findings and those reported by Howard et al. (8) in the development and preliminary validation of the Equine Brief Pain Inventory (EBPI). In both studies, the owner played a central role in the assessment of chronic pain, and subjective measures showed significant correlations with clinical and/or biomechanical parameters, supporting their potential usefulness for long-term clinical monitoring. In both cases, internal consistency analysis confirmed good reliability. However, there are also fundamental methodological differences: the EBPI is a structured, standardized tool with subscales for pain intensity and interference with daily activities, while the CSOM is tailored to the individual horse based on specific signs observed by the owner. Howard et al. (8) also performed convergent and discriminant validity tests against other validated instruments, while the present study focused on comparison with objective biomechanical measures (gait asymmetry) and VAS. Despite these differences, the convergence of results reinforces the idea that structured owner-based tools, if properly validated, can provide clinically relevant information that complements objective assessment methods.

Despite the small number of observations, the Bland–Altman analysis showed good agreement between pain assessments made by caretakers (VAS-own) and examiner (VAS-exp), with a low average bias, suggesting that on average caretakers provide similar scores as those attributed by the veterinarian. However, the relatively large Limits of Agreement (LOA) indicate some variability in individual assessments, with some significant discrepancies. This could be attributed to subjective factors, such as the caretaker’s experience, their perception of pain, the emotional bond with the animal and the placebo effect as already reported in other studies (29). The homogeneous distribution of the data around the mean bias suggests that the differences between the measurements do not depend on the intensity of the perceived pain but remain constant throughout the rating scale. Intraclass correlation analysis (ICC = 0.727, 95% CI) indicated good inter-rater reliability; however, agreement was moderate in the treatment group (T-group; ICC = 0.625) and higher in the control group (C-group; ICC = 0.838). These findings suggest that inter-rater agreement may vary according to group characteristics and treatment-related effects. The higher ICC observed in the Group C at inter-rater reliability evaluation between VAS-exp and VAS-own (good reliability) could be the expression of higher pain score in Group C. On the other hand, Group T showed a lower ICC (moderate reliability) and a higher interobserver variability (as demonstrated by the wider ICC confidence interval, and by the higher standard error, minimal detectable change and MDC% values). Analgesic treatment may have attenuated the ability to observe pain behaviour in Group T and thus could explain the lower interobserver ICC and higher 95%CI, SEM and MDC, compared to Group C. Such evidence reinforces the importance of proper training of all people involved in the assessment and treatment of pain (handlers, veterinarians, and caregivers). Such training could help standardize pain recognition across observers and reduce the risk of oligoanalgesia in horses.

From a methodological point of view, the evaluation of the internal consistency of the CSOM by means of the Cronbach’s alpha coefficient (0.809 in the total population, 0.890 in the C group and 0.787 in the T group) showed a good reliability of the instrument, although in the T group the greater clinical heterogeneity may have influenced the consistency of the evaluations. These data support the use of CSOMs as a repeatable and consistent method to monitor pain in horses affected by osteoarthritis over time.

Several limitations must be considered. Firstly, the lack of specific training for caretakers in the selection of the CSOM may have introduced bias into the data, with repetitive or overly generic choices (e.g., lameness). Furthermore, the small sample size (17 horses, with two excluded due to clinical worsening) limits the generalizability of the results. A longer observation period would be useful to better understand pain fluctuations over time, as osteoarthritis is a chronic condition characterized by exacerbation and remission phases. Environmental and individual factors may also have influenced the results. It is known that horses can alter pain expression depending on the presence of observers or external factors, such as variations in weather conditions also reported in human medicine. In addition, individual differences in the animal’s temperament could play a role in pain perception and response to treatment, as observed in other studies in which horses with greater neuroticism tended to exhibit greater sensitivity (30–33). Future studies should include larger cohorts, longer follow-up periods, and formal owner training sessions for the identification of CSOM items. Additionally, the recruitment of a control group of clinically healthy horses would allow the assessment of diagnostic performance parameters, including sensitivity, specificity, and receiver operating characteristic (ROC) curve analysis, thereby providing a more comprehensive evaluation of the discriminative ability of the CSOM. Furthermore, the integration of standardized reference scales for chronic pain in horses would substantially enhance the external validity and clinical applicability of CSOM in the equine field.

## 6 Conclusion

This study confirms that Client-Specific Outcome Measures (CSOM) are a potentially valuable tool for complementing the assessment of chronic osteoarthritis (OA) pain in horses, showing significant correlations with both subjective and objective measures. Analysis of the selected CSOM indicates that, beyond the typical locomotor impairments associated with OA, other pain dimensions—such as affective and motivational aspects—are also important to horse caretakers and need consideration while evaluating treatment success.

Overall, the good agreement between subjective assessments by caretakers and veterinary examiners highlights the potential role of the former in ongoing pain monitoring, especially in situations where frequent veterinary evaluations may be impractical.

## Data Availability

The original contributions presented in the study are included in the article/Supplementary material, further inquiries can be directed to the corresponding author.

## Footnotes

1
http://www.randomizer.org
