# Supplementary material for: Client-specific outcome measure for chronic osteoarthritis pain assessment in horses
Source: Front Vet Sci. 2026 Mar 23;13:1771745. doi: 10.3389/fvets.2026.1771745 (PMC13051653; doi:10.3389/fvets.2026.1771745)
Supplement: Supplementary file 2 [file Data_Sheet_2.pdf]

frontiers

Supplementary Material

1 Supplementary Tables (S1)

Horse	CSOM 1	CSOM 2	CSOM 3
H1	Non-physiologic locomotion	Pain on palpation of the affected joint	Prolonged resting of the limb
H2	Non-physiologic locomotion	Lifting contralateral limb	Walking to the pasture in the morning
H3	Activity level	Non-physiologic locomotion	Pain face
H4	Non-physiologic locomotion	Difficulty rising	Activity in the pasture
H5	Starting to walk / quality of first steps	Non-physiologic locomotion	Prolonged resting of the limb
H6	Non-physiologic locomotion	Pain face	Prolonged resting of the limb
H7	Overall impression an attitude	Non-physiologic locomotion	Motivation during work
H8	Motivation to walk on/forward	Non-physiologic locomotion	Motivation
H9	Non-physiologic locomotion	Reaction to flexion of the affected joint	Prolonged resting of the limb
H10	Prolonged resting of the limb	Non-physiologic locomotion	Camping under
H11	Non-physiologic locomotion	Motivation at work	Facial expression and attitude
H12	Non-physiologic locomotion	Stiffness when starting to walk	Flexion of the affected joint
H13	Non-physiologic locomotion	Motivation during the activity	Non-physiologic locomotion on a circle
H14	Shortened strides and walking slowly downhill	Non-physiologic locomotion	Prolonged resting of the limb
H15	Non-physiologic locomotion on a circle	Non-physiologic locomotion at trot	Non-physiologic locomotion on hard ground
H16	Non physiologic locomotion on a circle	Prolonged resting of the limb	Walking downhill
H17	Non-physiologic locomotion	Lifting contralateral limb	Stiffness when starting walking

Posture and weight-bearing	Motivation and activity	Evoked pain reaction	Facial expression

Supplementary S1. The table summarizes the different CSOM reported by the owners of the 17 horses (H1–H17). CSOM are color-coded and grouped into the four main categories identified in this study.
